# Impact of anemia treatment for left ventricular hypertrophy using long-acting erythropoietin-stimulating agents from the pre-dialysis to maintenance dialysis period in patients with chronic kidney disease, retrospective longitudinal cohort study

**DOI:** 10.1186/s12882-023-03133-1

**Published:** 2023-03-25

**Authors:** Hiroaki Io, Masahiro Muto, Yu Sasaki, Masanori Ishizaka, Toshiki Kano, Haruna Fukuzaki, Takuya Maeda, Yuki Shimizu, Junichiro Nakata, Yusuke Suzuki

**Affiliations:** 1 Department of Nephrology, Juntendo University Nerima Hospital, Takanodai 3-1-10, Nerima-ku, 177-8521 Tokyo, Japan; 2 Department of Nephrology, Faculty of Medicine, Juntendo University, Tokyo, Japan

**Keywords:** Renal anemia, Epoetin beta pegol, Left ventricular remodeling, Mean hemoglobin, Hemodialysis

## Abstract

**Background:**

Anemia in patients with chronic kidney disease (p-CKDs) may initiate or exacerbate left ventricular hypertrophy (LVH). This study aimed to determine whether treatment using long-acting erythropoietin-stimulating agents (L-ESAs) is independently associated with LVH during the pre-dialysis to maintenance dialysis period in p-CKDs.

**Methods:**

Physical and laboratory examinations were performed 120 days before initiating dialysis in p-CKDs (baseline). To evaluate the left ventricular mass index (LVMI) after starting dialysis, the mean hemoglobin (Hb) was defined as the average at the start of dialysis and 6 months after starting dialysis. Changes in the LVMI were observed in three groups according to mean Hb levels (Hb < 10.1, 10.1 < Hb < 11.0, and Hb > 11.0 g/dL for Groups 1, 2, and 3, respectively). LVMI was evaluated using echocardiography at the pre-dialysis, initiation, and maintenance dialysis periods.

**Results:**

A lower LVMI at dialysis initiation and an improvement in LVMI were detected in the highest tertile group of mean Hb (11.0 g/dl). Consequently, in the high Hb group (Hb level > 11.0 g/dl), LVMI remained low from dialysis initiation until after 6 months.The relationship between Hb and LVMI was not significant; however, a constant correlation with β ≥ 0.4 in the absolute value was maintained.

**Conclusion:**

L-ESAs may correlate with Hb and LVMI after administration, independent of the baseline LVMI and Hb values. These findings have therapeutic implications in the treatment strategies for p-CKDs during the pre-dialysis to maintenance dialysis period.

## Background

Cardiovascular disease is the primary reason for morbidity and mortality in patients with pre-dialysis chronic kidney disease (CKD) [1, 2]. Previously, we reported that the incidence of left ventricular hypertrophy (LVH) increased in direct correlation with the progression of CKD in patients with kidney failure [3]. The prevalence of LVH increases with the progression of kidney dysfunction. Before initiating dialysis, 85% of patients with stages 4 and 5 CKD undergo left ventricular (LV) remodeling [3]. LVH is recognized as a primary risk factor for cardiovascular death in patients undergoing dialysis [4]. It is a strong predictor of cardiac failure, sudden death, myocardial infarction, and stroke [5]. A previous study reported systolic blood pressure, residual glomerular filtration rate, and serum albumin (Alb) levels as predictive factors for LV mass index (LVMI), and these were measured using echocardiography at hemodialysis initiation [6]. Renal anemia is a common complication in patients with CKD (p-CKDs) [7]; it usually develops because of erythropoietin deficiency. In a previous study, the mean hemoglobin (Hb) value was found to be higher in an LVH-uncomplicated group than in an LVH-combined group from the pre-dialysis to dialysis initiation period when using long-acting erythropoietin-stimulating agents (L-ESAs) [8]. However, few longitudinal analyses have evaluated the factors associated with LVMI. Therefore, this retrospective cohort study investigated whether anemia treatment is independently associated with LVH during the pre-dialysis to maintenance dialysis period in p-CKDs using L-ESAs.

## Methods

### Study design and cohort

This retrospective longitudinal cohort study investigated 32 p-CKDs before dialysis initiation. The study protocol was in conformance with the ethical guidelines of our institution (Ethics Committee Approval No. 22–78 at Juntendo university Nerima hospital and No. 2015026 at Juntendo university) and was registered in the University Hospital Medical Information Network (UMIN; ID: UMIN000018312). Informed consent was obtained from each patient before they participated in the study. The criteria for enrollment in this study included no history of congestive heart failure (defined as dyspnea or interstitial edema on chest X-rays), valvular disease, LV systolic dysfunction with an ejection fraction less than 50%, arrhythmia, or abnormal electrocardiography. The study period was from July 2015 to July 2018. All patients underwent hemodialysis.

The physical and laboratory examinations and LVMI measured using echocardiography were performed three times (at baseline, dialysis initiation, and during the maintenance dialysis period). Regarding the data points, the first point (baseline) was when the patients were injected subcutaneously with L-ESAs, namely, epoetin beta pegol (*n* = 17) or epoetin beta (*n* = 15). The second point was when the patients were hospitalized to undergo preparation for dialysis (before starting dialysis, at the time of the creation of the arteriovenous fistula). The third point was at 6 months after dialysis initiation. Blood and urine samples were collected during the echocardiographic study. The clinical, laboratory, and urinary parameters of all patients were recorded at baseline, 6 months before starting dialysis, 3 months before starting dialysis, and at the start of dialysis. The patients were followed for 120 days or more from baseline to the start of dialysis to determine if L-ESAs were effective and stable in treating anemia and LV remodeling. The target Hb levels for the patients in the study were based on the Japanese Society for Dialysis Therapy treatment guidelines for renal anemia [9]. None of the patients were administered iron during the pre-dialysis period. To evaluate LVMI after the start of dialysis, the mean hemoglobin (Hb) was defined as the average at the start of dialysis and 6 months after starting dialysis. The patients were divided into tertile groups based on the mean Hb level during the observation period.

### Physical and laboratory examinations

Blood pressure (BP) was measured using a manual sphygmomanometer with the patient in a sitting position either after 5 min of rest before echocardiography or at regular visits to the outpatient clinic. For the study parameters, we selected traditional risk factors that have been reported to be closely associated with CKD and LVH. The laboratory parameters were as follows: serum creatinine, serum Alb, Hb, correct serum calcium (cCa), serum phosphorus (P), intact parathyroid hormone, brain natriuretic peptide (BNP), high-sensitivity C-reactive protein, and ferritin levels as well as transferrin saturation (TSAT). The cCa was calculated as follows: cCa = Ca + (4-Alb)]. The TSAT was calculated as follows: serum iron/total iron-binding capacity) × 100 (%). The erythropoietin resistance index (ERI; a dosage of epoetin/body weight/Hb level) was also calculated. ESA doses were converted to epoetin doses using dose conversion ratios (epoetin beta:epoetin beta pegol = 200:0.93) [10]. The serial course of Hb levels and ERI was compared with the amount of epoetin beta pegol used during each observation period.

### Echocardiography

For all patients, echocardiography (two-dimensional and M-mode measurement) was performed using LOGIQe with a 2.5 MHz phased array transducer from GE Yokogawa Medical Systems (Tokyo, Japan). Echocardiographic examinations were conducted on a non-hemodialysis day. All echocardiographic data were evaluated according to the American Society of Echocardiography guidelines [11]. The left atrial and LV size, intraventricular septal thickness, posterior LV wall thickness (PWT), and LV mass were recorded [12]. LVMI was used to express the LV mass, which was corrected using the body surface area [13]. The relative wall thickness (RWT) was calculated as follows: 2 × PWT/LV diastolic diameter (LVDd). The severity of LVH was thus assessed based on LVMI and RWT.

### Statistical analyses

We consulted Statista (Hamburg, Germany) for statistical analyses. This was a pilot study, and the number of cases was limited; thus, we did not correct the multiplicity of the tests. All data are expressed as means ± standard deviations. The Student’s *t*-test was used for the univariate analysis. Variables with *p*-values < 0.05 were analyzed using a stepwise linear regression analysis based on a forward–backward procedure. The F-value for entry or removal of candidate variables from the discriminant function was set at 4.0.

## Results

### Patient characteristics

**Fig. 1:**
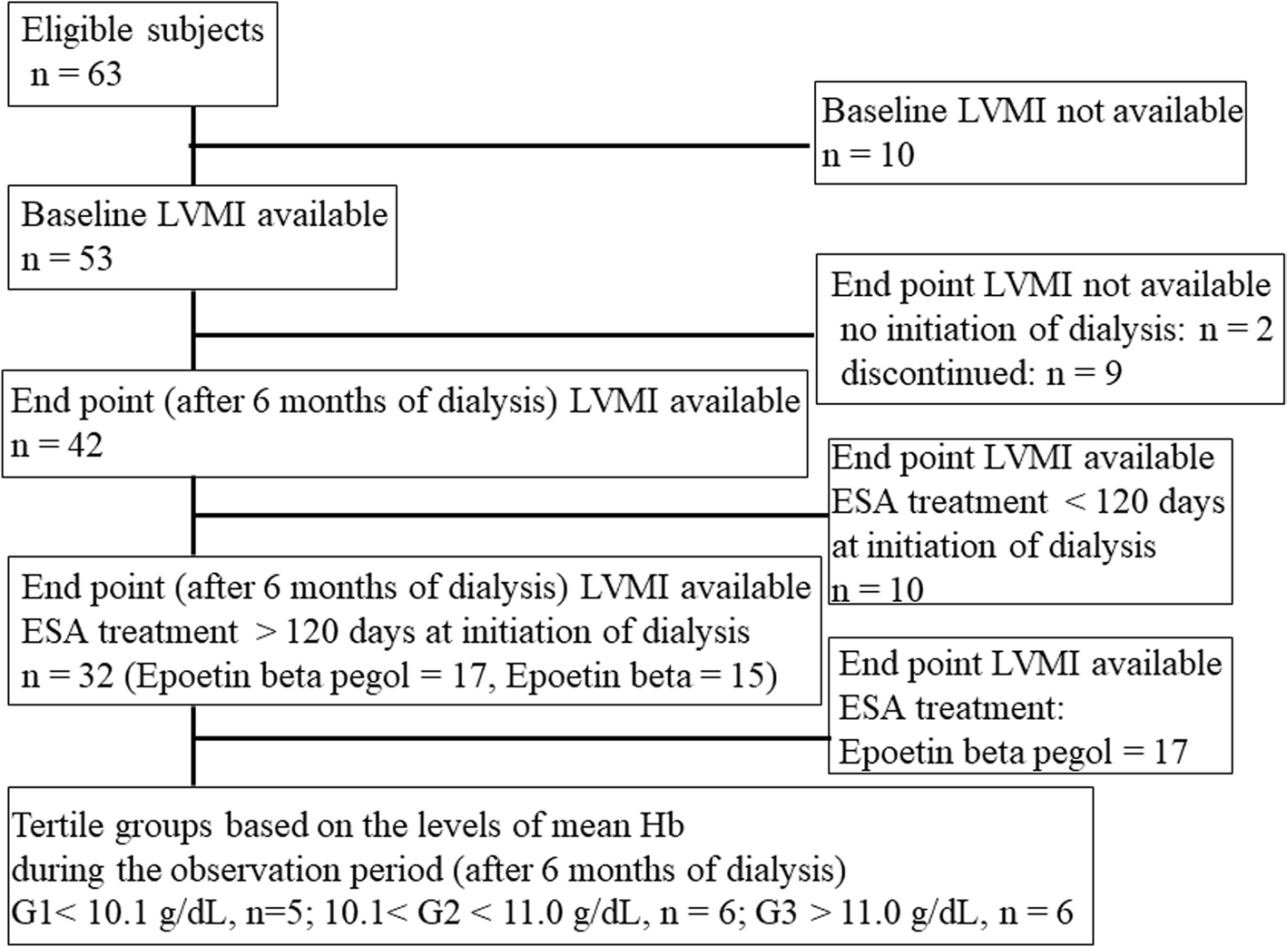
Patient disposition and flow chart showing patient selection criteria. *LVMI* Left ventricular mass index, *ESA* Erythropoietin-stimulating agents, *Hb* Hemoglobin, G1, Group 1; G2, Group 2; Group 3

A flow chart depicting the patient selection criteria is shown in Fig. 1. The patients were divided into three groups based on their mean Hb levels during the observation period as follows: Group 1 (G1), Hb < 10.1 g/dL; Group 2 (G2), 10.1 < Hb < 11.0; and Group 3 (G3), Hb > 11.0 g/dL. No significant differences were observed between the groups in terms of baseline patient characteristics, except for the use of epoetin beta pegol (MIRCERA), primary renal disease (percentage of diabetic patients with primary renal disease), and urinary protein excretion (Table 1).

**Table 1:** Patients’ characteristics

	Average Hb < 10.1			10.1 ≤ Average Hb < 11.0			11.0 ≤ Average Hb		
	Baseline	At dialysis	Endpoint	Baseline	At dialysis	Endpoint	Baseline	At dialysis	Endpoint
N	5			6			6		
Term before dialysis (days)	340.8 ± 32.8			283.7 ± 132.0			251.7 ± 221.4		
Age (years old)	71.8 ± 10.7			62.7 ± 11.2			56.3 ± 13.4		
Sex (n, (% men))	5, (100.0)			4, (66.7)			4, (66.7)		
Diabetes (n, %)	4, (80.0)			4, (66.7)			5, (83.3)		
Weight (kg)	59.2 ± 13.4	60.4 ± 14.4	58.0 ± 11.5	68.8 ± 10.4	69.0 ± 10.8	67.0 ± 10.8	64.5 ± 18.2	65.2 ± 17.3	61.0 ± 12.5
SBP (mmHg)	138.0 ± 14.0	141.2 ± 19.7	137.6 ± 17.8	143.0 ± 13.5	143.0 ± 11.5	142.0 ± 10.1	134.0 ± 9.9	128.7 ± 17.4	139.2 ± 11.1
DBP (mmHg)	76.5 ± 5.7	75.6 ± 12.7	73.2 ± 20.0	73.5 ± 14.3	72.7 ± 11.0	72.0 ± 15.9	82.5 ± 14.5	70.3 ± 9.9	85.2 ± 4.1
Hemoglobin (g/dL)	9.6 ± 0.8	9.7 ± 0.7	10.1 ± 0.8	9.5 ± 1.4	9.9 ± 0.7	10.9 ± 0.5#	9.9 ± 0.8	10.9 ± 0.8*	12.2 ± 0.8*, **, #
ERI	42.0 ± 32.6	37.1 ± 16.8	33.1 ± 5.2	32.7 ± 21.8	26.8 ± 14.7	29.8 ± 19.4	36.1 ± 20.1	37.4 ± 20.7	23.6 ± 20.8##
TSAT (%)	34.5 [30.1, 45.0]	23.7 [19.4, 43.7]	20.4 [16.9, 21.2]	32.8 [29.7, 36.2]	17.2 [11.6, 38.5]	31.6 [24.8, 45.0]*	32.8 [23.3, 44.4]	17.1 [12.6, 23.1]#	29.6 [22.2, 62.7]
Ferritin (mg/dL)	290 [121, 507]	176 [69, 256]	231 [69, 265]	139 [107, 341]	193 [62, 537]	247 [125, 361]	140 [103, 271]	122 [68, 310]	172 [97, 442]
BNP (pg/mL)	74 [32, 163]	482 [106, 1130]	648 [67, 1189]	138 [62, 177]	47 [18, 409]	269 [153, 343]	26 [19, 34]	41 [21, 88]	50 [19, 191]
Intact PTH (mg/dL)	219 [161, 700]	215 [115, 548]	320 [156, 648]	406 [139, 817]	237 [15, 673]	141 [93, 282]	287 [283, 722]	220 [65, 485]	161 [93, 320]
Correction Ca (mg/dL)	8.7 ± 0.2	9.1 ± 0.9	9.3 ± 0.7#	8.8 ± 0.4	8.7 ± 0.3	8.6 ± 0.2	8.6 ± 0.5	9.4 ± 0.9	9.5 ± 0.8
Phosphorus (mg/dL)	4.2 ± 1.3	5.1 ± 1.3	5.7 ± 1.1#	4.4 ± 0.5	5.0 ± 1.2	5.0 ± 1.5	5.7 ± 0.5**	6.0 ± 1.2	5.7 ± 1.7
Albumin (mg/dL)	3.8 ± 0.4	3.3 ± 0.5	2.9 ± 0.7#	4.0 ± 0.5	3.6 ± 0.4	3.6 ± 0.5	4.0 ± 0.4	3.7 ± 0.4	3.5 ± 0.4
hsCRP (mg/dL)	0.07 [0.03, 0.10]	0.03 [0.01, 0.36]	0.03 [0.01, 0.14]	0.28 [0.15, 0.40]	0.14 [0.09, 0.80]	0.05 [0.03, 0.06]	0.09 [0.08, 0.10]	0.11 [0.06, 5.95]	0.16 [0.06, 0.30]
Creatinine (mg/dL)	4.7 ± 1.7	9.3 ± 3.4#	-	5.7 ± 2.1	8.7 ± 1.9#	-	7.0 ± 0.9*	10.3 ± 1.9#	-
Urinary protein (g/g Cr)	2.3 ± 1.0	3.9 ± 4.5	-	2.6 ± 1.9	1.9 ± 0.6	-	1.4 ± 1.1	1.5 ± 0.7	-
Echocardiography									
LVDd (mm)	52.0 ± 0.1	51.7 ± 3.4	55.7 ± 7.5	49.4 ± 7.2	48.9 ± 4.8	47.7 ± 3.8	49.8 ± 1.1	44.6 ± 4.9*	46.1 ± 4.3*
Ejection fraction (%)	0.61 ± 0.19	0.60 ± 0.12	0.58 ± 0.10	0.70 ± 0.15	0.67 ± 0.13	0.67 ± 0.11	0.67 ± 0.01	0.71 ± 0.06	0.64 ± 0.09##
LVMI (g/m^2^)	163.5 ± 39.6	162.7 ± 45.7	205.9 ± 41.5	143.3 ± 45.3	143.1 ± 32.8	149.7 ± 25.8*	117.6 ± 17.7*	110.5 ± 31.1	123.2 ± 29.7*
RWT	0.41 ± 0.02	0.48 ± 0.06	0.44 ± 0.06	0.45 ± 0.07	0.48 ± 0.04	0.50 ± 0.06	0.43 ± 0.04	0.49 ± 0.05#	0.48 ± 0.07
Treatment (n, (%))									
ARB	2, (40.0)	1, (20.0)	1, (20.0)	1, (16.7)	1, (16.7)	2, (33.3)	2, (33.3)	3, (50.0)	3, (50.0)
CCB	1, (20.0)	1, (20.0)	1, (20.0)	0, (0.0)	0, (0.0)	1, (16.7)	3, (50.0)	3, (50.0)	4, (66.7)
β-blocker	4, (80.0)	4, (80.0)	3, (60.0)	4, (66.7)	3, (50.0)	2, (33.3)	5, (83.3)	5, (83.3)	5, (83.3)

Abbreviations: *SBP* Systolic blood pressure, *DBP* Diastolic blood pressure, *ERI* Erythropoietin resistance index, *TSAT* Transferrin saturation, *BNP* brain natriuretic peptide, *PTH* Parathyroid hormone, *Ca* Calcium, *hsCRP* High-sensitivity C-reactive protein, *LVDd* Left ventricular diastolic dysfunction, *LVMI* Left ventricular mass index, *RWT* Relative wall thickness. Average Hb: average at baseline and dialysis

^*^ *p* < 0.05 vs. Average Hb < 10.1, **p < 0.05 vs. 10.1 ≤ Average Hb < 11.0 (unpaired test: Fisher’s exact test [nominal scale], t-test [continuous scale and parametric], Mann–Whitney U test [continuous scale and non-parametric])

^#^ *p* < 0.05 vs. Baseline, ##*p* < 0.05 vs. At dialysis (paired test: paired t-test [continuous scale and parametric], Wilcoxson signed-rank test [continuous scale and non-parametric])

### Evaluation of LV remodeling at follow-up using LVMI

**Fig. 2:**
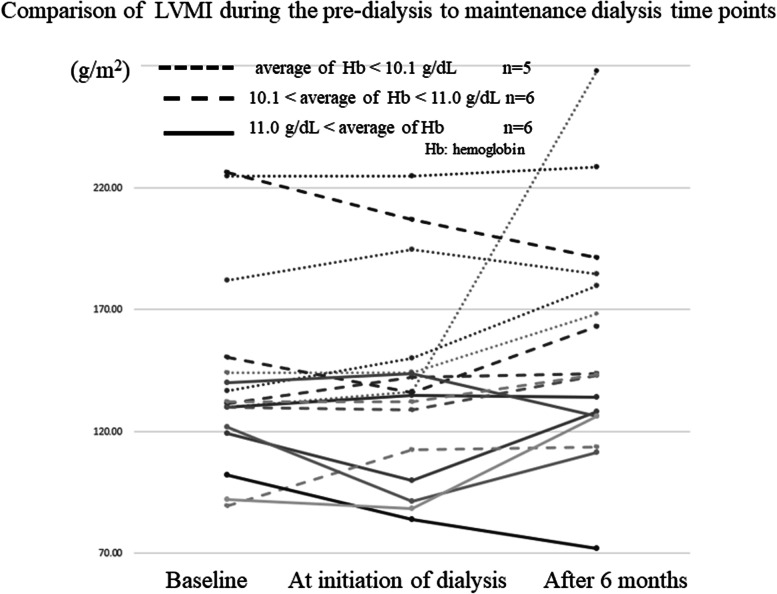
Comparison of the left ventricular mass index at the pre-dialysis to maintenance dialysis periods. *Hb* Hemoglobin

A comparison of LVMI at the baseline, initiation, and after 6 months is shown in Fig. 2. We detected a lower LVMI at baseline (pre-dialysis period) along with improvements in the LVMI during the observation period in G3 (> 11.0 g/dl). The LVMI of the patients in G3 (> 11.0 g/dl) was significantly lower than that of the other groups. The LVMI showed no difference at the start of ESA administration and dialysis initiation between the groups using epoetin beta pegol and epoetin beta (data not shown, see Reference 8). Changes in LVMI were observed in the three groups according to the mean Hb level from the start of ESA administration to dialysis initiation. As a result, in the high Hb group, where the Hb level exceeded 11.0 g/dl, the LVMI remained low from the time of dialysis initiation to after 6 months (Fig. 2).

### Factors associated with LVMI during the observation period

As shown in Table 1, there was a clear difference in LVMI after 6 months. Table 2 shows the results from the univariate linear regression analysis of factors associated with LVMI after 6 months of dialysis. The LVMI after 6 months was significantly associated with age, male sex, Hb, TSAT, and mean Hb. Table 3 shows the results from the multivariate linear regression analysis of factors associated with LVMI after 6 months of dialysis. In Model 1, age and sex were significant factors; however, in Model 2, the relationship between Hb and LVMI was not significant, and a constant correlation with β ≥ 0.4 in absolute value was maintained. This suggested that Hb and LVMI after continuous administration of erythropoiesis receptor activator might be correlated independent of the initial LVMI and Hb values.

**Table 2:** Univariate linear regression analysis of factors associated with LVMI

	LVMI after 6 months of dialysis (univariate analysis)			
	Regression coefficients	Standard error	β	*p*-value
Age	1.941	0.776	0.543	**0.024**
Sex: male	56.234	22.901	0.535	**0.027**
Baseline Hb	-15.131	11.080	-0.333	0.192
After 6 months of dialysis				
Weight	-0.599	1.080	-0.147	0.588
Systolic blood pressure	-0.414	1.055	-0.108	0.701
Diastolic blood pressure	-1.112	0.825	-0.350	0.201
Hemoglobin	-26.910	8.466	-0.634	**0.006**
ERI	-0.249	0.738	-0.090	0.741
TSAT	-191.513	81.409	-0.597	**0.040**
Ferritin	-0.089	0.117	-0.235	0.462
BNP	0.130	0.028	0.840	**0.001**
Intact PTH	0.026	0.087	0.094	0.771
Correct calcium	-28.354	29.733	-0.319	0.368
Phosphorus	-2.779	15.001	-0.065	0.858
Mean Hb	-30.969	13.765	-0.502	**0.040**

β: Standardized regression coefficients

Abbreviations: *BNP* Brain natriuretic peptide, *PTH* Parathyroid hormone, *hsCRP* High-sensitivity C reactive protein, *TSAT* Transferrin saturation, *Hb* Hemoglobin, *ERI* Erythropoietin resistance index, *LVMI* Left ventricular mass index

**Table 3:** Multivariate linear regression analysis of factors associated with LVMI

	LVMI after 6 months of dialysis (multivariate analysis)			
	Regression coefficients	Standard error	β	*p*-value
Model 1: Age, Sex adjusted				
Hb of After 6 months	-15.557	7.721	-0.367	0.065
Age	1.683	0.574	0.471	**0.012**
Sex	38.335	18.720	0.365	0.061
Model 2: Baseline Hb and LVMI adjusted				
Hb of After 6 months	-18.077	9.537	-0.426	0.080
Baseline LVMI	0.546	0.368	0.460	0.161
Baseline Hb	3.765	12.570	0.083	0.769

β: Standardized regression coefficients

*LVMI* Left ventricular mass index, *Hb* Hemoglobin

### Serial processing of Hb levels and ERI

**Fig. 3:**
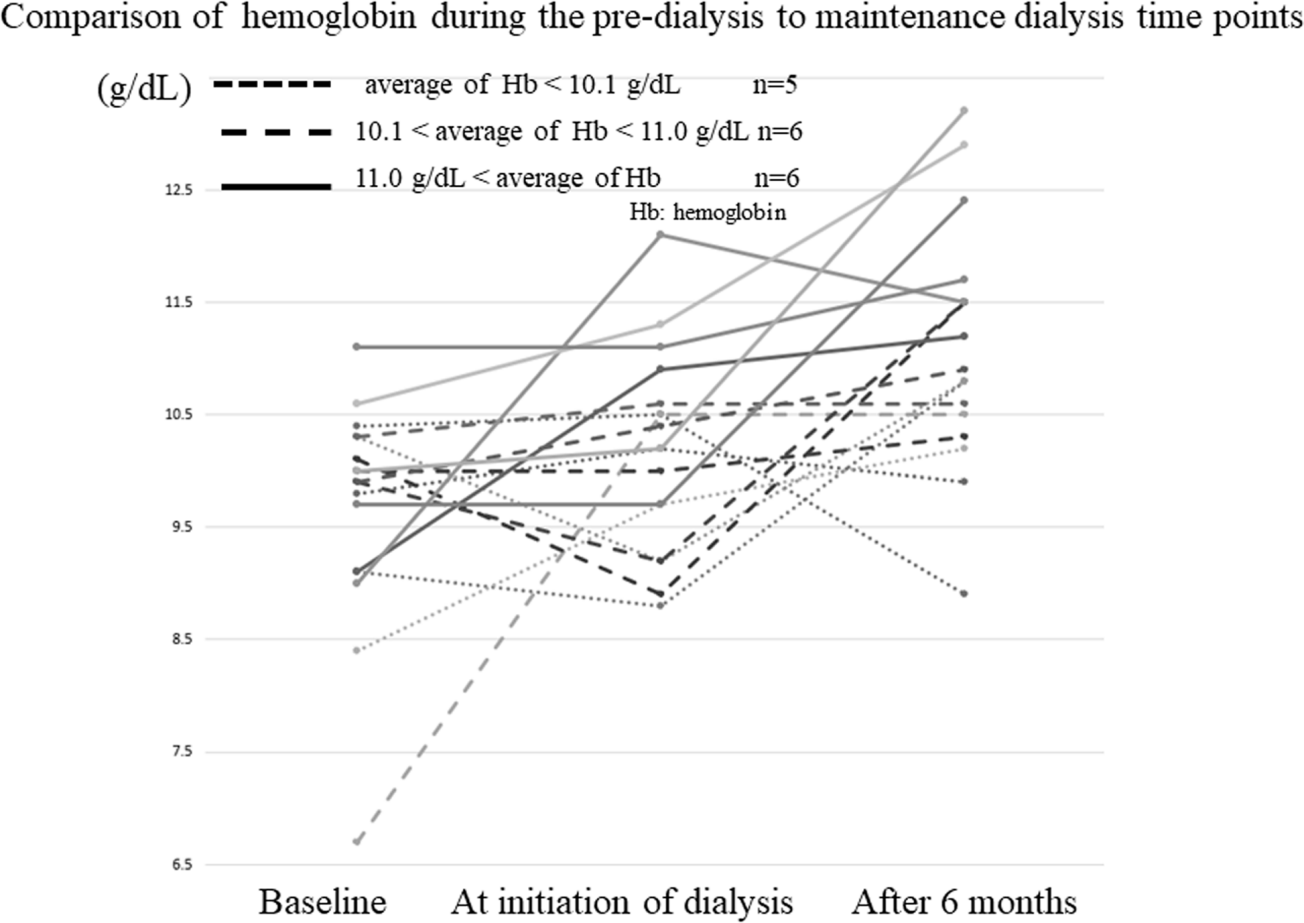
Comparison of hemoglobin levels during the pre-dialysis to maintenance dialysis period. Serial process of levels of hemoglobin

**Fig. 4:**
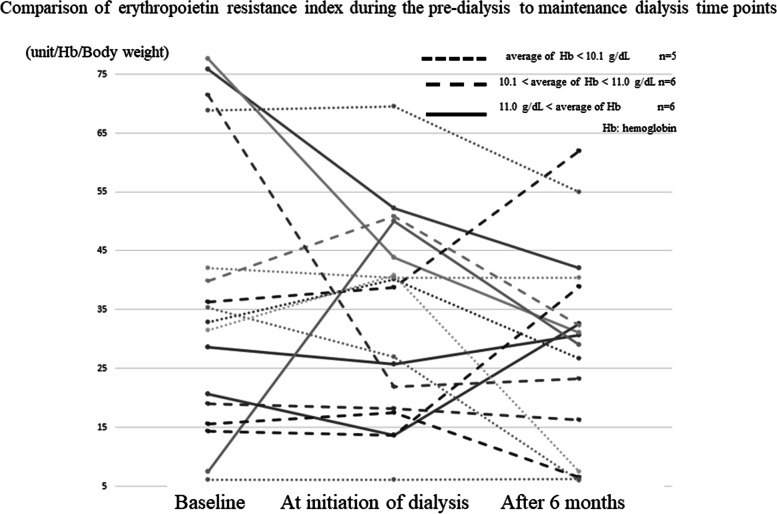
Comparison of the levels of erythropoietin resistance index during the pre-dialysis to maintenance dialysis periods. Serial process of levels of erythropoietin resistance index

When epoetin beta pegol was used, Hb levels after 6 months of dialysis were significantly higher than those at baseline and the start of dialysis (Fig. 3). Hb levels at dialysis initiation were lower than baseline Hb levels in the group using short-acting ESA (S-ESA) (epoetin beta) (data not shown, see Reference 8). The ERI after 6 months of dialysis was lower than that at baseline and dialysis initiation in patients using L-ESAs (Fig. 4).

The ERI at dialysis initiation and 3 months prior was significantly higher than that at baseline in the group using S-ESA (data not shown, see Reference 8). Patients using L-ESAs showed a significant improvement in ERI compared with patients using S-ESA in the multivariate analysis of variance (ANOVA) (data not shown, see Reference 8).

## Discussion

We aimed to determine whether treatment using long-acting erythropoietin-stimulating agents (L-ESAs) is independently associated with LVH during the pre-dialysis to maintenance dialysis period in p-CKDs. We found that L-ESAs were effective and stable when treating anemia until the maintenance dialysis period. Anemia is a causative factor for LVH in p-CKDs, and studies have shown correlations between age, anemia, LV mass, and CKD [3]. Conversely, anemia and LVH have indicated associations with declining renal function in p-CKDs; the interactions among aging, anemia, LVH, and CKD are complex [7, 14]. LVH is an independent risk factor for cardiac death in p-CKDs and end-stage renal disease. Severe CKD is associated with a higher prevalence of and more severe LVH. Interestingly, an elevated LV mass and LV geometry pattern appear to be important in predicting the response to anemia correction in p-CKDs. Previous reports have shown that eccentric hypertrophy is a greater risk factor than concentric hypertrophy for poor cardiovascular outcomes and worsening renal function in anemic p-CKDs [15, 16]. In this study, the measurement of the LVDd decreased upon initiating dialysis (Table 1). There was no significant difference in RWT throughout the observation period, which indicated that the patients in our study had concentric LVH. Several studies have examined the value of aggressively treating p-CKDs with recombinant erythropoietin to prevent the development of LVH [16]. Possible explanations for this finding include increased BP and blood viscosity in the treated patients. The Alb, urinary proteins, BNP, and BP levels from this study were neither significantly correlated nor independently associated with LVMI, as seen in the multivariate regression analysis (Tables 2 and 3). We believe that anemia causes high cardiac output to normalize wall stress, causing LV dilation due to increased preload followed by compensatory hypertrophy. There was no significant difference among the groups in terms of BP (Table 1).

Treating anemia improves survival, thereby decreasing morbidity and mortality and improving the quality of life in p-CKDs. A previous study reported that LVMI decreased due to increased Hb levels [17]. Our results are consistent with the findings from previous studies evaluating p-CKDs [3, 6, 7]. However, the Correction of Hemoglobin and Outcome in Renal Insufficiency study revealed that a targeted Hb level of 13.5 g/dl was more harmful than 11.3 g/dl in pre-dialysis p-CKDs and resulted in no incremental improvement in the quality of life [18]. The Cardiovascular Reduction Early Anemia Treatment Epoetin β study showed that with mild-to-moderate anemia in p-CKDs, the normalization of Hb levels in the range of 13.0–15.0 g/dl did not reduce cardiovascular events compared with the effects of achieving a lower target range (10.5–11.5 g/dl) [19]. Evidence regarding the target value for anemia treatment in p-CKDs is insufficient, especially for the upper limit target value, and further research is warranted. In this study, the Hb level reached the target value. For such cases, aggressive treatment (nephrologist follow-up; patients with a history of nephropathy treatment) is considered necessary [8]. In an observational study, the conversion from epoetin beta to epoetin beta pegol maintained a Hb level between 11.2 g/dl and 11.4 g/dl after 12 months [20]. Our previous study showed that the treatment of anemia prevented LVH, and the use of L-ESAs led to a more significant improvement in the ERI than using S-ESA in a few instances, as was recorded in the multivariate ANOVA analysis [8].

A previous study indicated that older age, high body mass index, pretreatment for Hb, use of angiotensin conversion enzyme/angiotensin receptor blocker, and diabetic nephropathy were associated with increased erythropoietin requirements in anemic p-CKDs [21]. In this study, most patients had diabetic nephropathy in the lowest tertile of mean Hb (< 10.1 g/dl). In p-CKDs with a history of nephropathy treatment who were not referred to a nephrologist, the risks of end-stage renal disease were higher in those with stages 3b-5 CKD [22]. Previous reports have shown that anemia and resistance to ESAs are prognostic factors in hemodialysis patients [23]. In addition, an association was found between Hb levels and the cardiothoracic ratio in patients on incident dialysis [24]. These reports partly supported our results.

This study has a few limitations. First, the duration of the observation periods was different among the patients. Additionally, this was a pilot study and the number of cases was limited; thus, we did not correct the multiplicity for the tests. Second, echocardiography data were examined using only three data points. We could have established a more accurate difference if five or six points had been used. An increase in LVMI is a prerequisite for developing LVH. Accurate echocardiographic screening of p-CKDs is an available tool to rule out the presence of LVH. Third, all the patients had different personal, medical, and treatment histories. In the future, studies with larger sample sizes and comprising patients with similar characteristics need to be performed to determine the outcomes of this treatment more accurately. Moreover, the results of comparative studies between ESA and hypoxia-inducible factor-prolyl hydroxylase inhibitors are expected.

In conclusion, using L-ESAs appears to be an effective and stable method of treating anemia until after 6 months of dialysis. Therefore, it is essential to treat anemia to prevent LV remodeling in p-CKDs. These findings may have therapeutic implications for treatment strategies in p-CKDs with L-ESAs administered during the pre-dialysis phase until maintenance dialysis.

## Data Availability

The datasets used and/or analysed during the current study are available from the corresponding author on reasonable request.

## Abbreviations

ANOVA: Analysis of variance
BNP: Brain natriuretic peptide
BP: Blood pressure
CKD: Chronic kidney disease
ESA: Erythropoietin-stimulating agents
ERI: Erythropoietin resistance index
LV: Left ventricular
LVH: Left ventricular hypertrophy
LVMI: LV mass index
PWT: Posterior LV wall thickness
RWT: Relative wall thickness
TSAT: Transferrin saturation

## References

[1] Go AS, Chertow GM, Fan D, McCulloch CE, Hsu CY (2004). Chronic kidney disease and the risks of death, cardiovascular events, and hospitalization. N Engl J Med.

[2] Vanholder R, Massy Z, Argiles A, Spasovski G, Verbeke F, Lameire N (2005). Chronic kidney disease as cause of cardiovascular morbidity and mortality. Nephrol Dial Transplant.

[3] Matsumoto M, Io H, Furukawa M, Okumura K, Masuda A, Seto T (2012). Risk factors associated with increased left ventricular mass index in chronic kidney disease patients evaluated using echocardiography. J Nephrol.

[4] Foley RN, Parfrey PS, Kent GM, Harnett JD, Murray DC, Barre PE (1998). Long-term evolution of cardiomyopathy in dialysis patients. Kidney Int.

[5] Levy D, Garrison RJ, Savage DD, Kannel WB, Castelli WP (1990). Prognostic implications of echocardiography determined left ventricular mass in the Framingham Heart Study. N Engl J Med.

[6] Io H, Matsumoto M, Okumura K, Sato M, Masuda A, Furukawa M (2011). Predictive factors associated with left ventricular hypertrophy at baseline and in the follow-up period in non-diabetic hemodialysis patients. Semin Dial.

[7] Okumura K, Io H, Matsumoto M, Seto T, Takagi M, Masuda A (2013). Predictive factors associated with change rates of LV hypertrophy and renal dysfunction in CKD patients. Clin Nephrol.

[8] Io H, Aizawa M, Funabiki K, Horikoshi S, Tomino Y (2015). Impact of anaemia treatment for left ventricular remodelling prior to initiation of dialysis in chronic kidney disease patients: Efficacy and stability of long acting erythropoietin stimulating agents. Nephrology (Carlton).

[9] Yamamoto H, Nishi S, Tomo T, Masakane I, Saito K, Nangaku M (2017). Japanese Society for Dialysis Therapy: Guidelines for renal anemia in chronic kidney disease. Ren Replace Ther.

[10] Kuwahara M, Hasumi S, Mandai S, Tanaka T, Shikuma S, Akita W (2014). Effects of three kinds of erythropoiesis-stimulating agents on renal anemia in Japanese non-dialysis chronic kidney disease patients. Clin Exp Nephrol.

[11] Lang RM, Bierig M, Devereux RB, Flachskampf FA, Foster E, Pellikka PA (2005). Recommendations for chamber quantification: A report from the American Society of Echocardiography’s Guidelines and Standards Committee and the Chamber Quantification Writing Group, developed in conjunction with the European Association of Echocardiography, a branch of the European Society of Cardiology. J Am Soc Echocardiogr.

[12] Devereux RB, Alonso DR, Lutas EM, Gottlieb GJ, Campo E, Sachs I (1986). Echocardiographic assessment of left ventricular hypertrophy: Comparison to necropsy findings. Am J Cardiol.

[13] Harnett JD, Murphy B, Collingwood P, Purchase L, Kent G, Parfrey PS (1993). The reliability and validity of echocardiographic measurement of left ventricular mass index in hemodialysis patients. Nephron.

[14] Chang JM, Chen SC, Huang JC, Su HM, Chen HC (2014). Anemia and left ventricular hypertrophy with renal function decline and cardiovascular events in chronic kidney disease. Am J Med Sci.

[15] Moon SJ, Bae KS, Park HC, Kim JK, Park JT, Lee JE (2011). The effect of anemia and left ventricular geometric patterns on renal disease progression in type 2 diabetic nephropathy. J Nephrol.

[16] Eckardt KU, Scherhag A, Macdougall IC, Tsakiris D, Clyne N, Locatelli F (2009). Left ventricular geometry predicts cardiovascular outcomes associated with anemia correction in CKD. J Am Soc Nephrol.

[17] Hayashi T, Suzuki A, Shoji T, Togawa M, Okada N, Tsubakihara Y (2000). Cardiovascular effect of normalizing the hematocrit level during erythropoietin therapy in predialysis patients with chronic renal failure. Am J Kidney Dis.

[18] Singh AK, Szczech L, Tang KL, Barnhart H, Sapp S, Wolfson M (2006). Correction of anemia with epoetin alfa in chronic kidney disease. N Engl J Med.

[19] Drüeke TB, Locatelli F, Clyne N, Eckardt KU, Macdougall IC, Tsakiris D (2006). Normalization of hemoglobin level in patients with chronic kidney disease and anemia. N Engl J Med.

[20] Padullés-Zamora N, Comas-Sugrañes D, Pineda-Yuste Mdel M, Jódar-Masanés R, Martínez-Castelao A (2012). Use of methoxy polyethylene glycol-epoetin beta in stage 3, 4 or 5 non-dialysis chronic kidney disease. Nefrologia.

[21] Rossert J, Gassmann-Mayer C, Frei D, McClellan W (2007). Prevalence and predictors of epoetin hyporesponsiveness in chronic kidney disease patients. Nephrol Dial Transplant.

[22] Minutolo R, Lapi F, Chiodini P, Simonetti M, Bianchini E, Pecchioli S (2014). Risk of ESRD and death in patients with CKD not referred to a nephrologist: A 7-year prospective study. Clin J Am Soc Nephrol.

[23] Panichi V, Rosati A, Bigazzi R, Paoletti S, Mantuano E, Beati S (2011). Anaemia and resistance to erythropoiesis-stimulating agents as prognostic factors in haemodialysis patients: Results from the RISCAVID study. Nephrol Dial Transplant.

[24] Asakawa T, Joki N, Tanaka Y, Hayashi T, Hase H, Komatsu Y (2014). Association between the hemoglobin level and cardiothoracic ratio in patients on incident dialysis. Cardiorenal Med.

